# Access to chronic medicines: patients’ preferences for a last kilometre medicine delivery service in Cape Town, South Africa

**DOI:** 10.1186/s12875-021-01392-1

**Published:** 2021-02-22

**Authors:** Siraaj Adams, Mwila Mulubwa, Mea van Huyssteen, Angeni Bheekie

**Affiliations:** 1 Iyeza Health, 4 Phatha Close, Harare Business Square, Khayelitsha, Cape Town, South Africa; 2 School of Pharmacy, University of the Western Cape, Private bag X17, Bellville, Cape Town, South Africa

**Keywords:** Chronic medicine, Differentiated service delivery models, Last kilometre, Medicine delivery, Patient preference

## Abstract

**Background:**

Chronic patients are required to access their chronic medicines on a regular basis, often only to refill their repeat prescriptions. Adherence to chronic medicines is challenging and has stimulated health care providers to devise differentiated service delivery models of care to decentralise chronic medicine distribution to decrease the frequency of medicine collection at health care facilities. One such option includes a last kilometre medicine delivery service. This study investigated chronic patients’ preferences for a last kilometre medicine delivery service model.

**Methods:**

An exploratory non-randomised quantitative study was conducted over 4 weeks at four public sector primary health care facilities in Cape Town, South Africa. Data was collected on a structured questionnaire from chronic patients queuing to receive medication at each facility’s pharmacy waiting area. Patient demographics were noted to align with preferences for chronic medicine service delivery characteristics including; mobile ordering, fee for service and location for delivery. Chi-square test and frequencies were employed to analyse data using SPSS version 23.

**Results:**

A total of 116 patients participated in this study. Most were interested in a medicine delivery service (80.2%) and were willing to use a mobile application to order their medicines (84.5%). Almost all patients (96.8%) preferred that their medicines be delivered to their home. More than three quarters of participants were willing to pay for the service (77.6%). Chi-square test showed that gender, age group, employment status, distance to the health facility and /or average waiting time at the clinic significantly influenced the preference for certain characteristics of the medicine delivery service (*p* < 0.05).

**Conclusion:**

Most participants were interested in a last kilometre medicine delivery service, especially those older than 45 years, waiting for more than 6 h at the facility, and staying within one kilometre radius of the clinic. More studies are needed to establish the influence of patients’ employment status and the distance to health facility on interest in the medicine delivery service.

**Supplementary Information:**

The online version contains supplementary material available at 10.1186/s12875-021-01392-1.

## Background

Chronic patients have to access their chronic medicines on a regular basis, often attending health care facilities only to refill their chronic prescriptions. A consistent supply and collection of medicines is important for adherence among chronic patients in order to decrease morbidity and mortality from chronic illnesses [1–3]. The patient’s long-term adherence is influenced by patient-related factors, the disease condition, medicine therapy, socio-economic situation and the health system [4].

Most interventions addressing chronic medicine adherence, at least in developing contexts, are from antiretroviral programmes [5], although there has been some investigations into non-communicable diseases (NCDs) [6]. These interventions focussed on differentiated service delivery (DSD) models of care to facilitate adherence to chronic medicine treatment [6–8]. Such service models have been divided into characteristics such as group versus individual interventions, facility versus community based, and health care provider and patient-based interventions for ARV treatment [7, 9]. The primary interventions for NCDs include primarily individual-based interventions, such as centralised dispensing with or without, less frequent facility visits and/or decentralised medicine collection points [1, 6].

The development of the DSD models was largely based on socio-economic factors impacting on the patients’ ability to access health care and medicines [10, 11]. Even though public sector facilities in South Africa have no fee for services or medicines, indirect costs such as transport and time at the facility does disadvantage the poorest patients [12], thereby prompting decentralisation of care and medicine supply points [13] and consequently, decreased facility contact visits. A recent systematic review found that most evidence for successful DSD interventions were for multi-month prescribing [7, 14], which implies less frequent facility visits.

DSD interventions have focussed on strengthening/streamlining the health system and have often generalised interventions to decongest the health system and decrease workload [6, 8]. In developing countries where health systems are stretched and under resourced, communication with patients regarding acceptability and affordability of health services is often overlooked in favour of stock availability and workload [15]. Yet, a literature review on ART adherence clubs in the Western Cape, South Africa found that community embeddedness which encouraged patient empowerment and participation was a strong enabler for this model’s sustainability [16].

However, evidence for DSD interventions was often based on intervention versus traditional care and not on their comparative desirability among patients [17]. Indeed, amongst the few DSD investigations that compared desirability of different models, patient preference was strongest for decreased clinic visits, and, individual and facility-based interventions in urban areas [17]. There seems to be increased consensus with investigations into DSD models that multiple interventions [9] that address patient, socio-economic and health system needs, should enable patients to make their preferred choices [15, 16, 18].

Few interventions have investigated last kilometre delivery of medicines [7], which begs the question, what would chronic patients prefer in terms of a last kilometre delivery service for their medicines? The global health community frequently invokes the concept of the “last mile” or “last kilometre” to refer to achieving coverage for those clients who are the most difficult to reach” [19] (Pg 511). In this study reference to the last kilometre was literal, i.e. getting the medicines into the hands of the patient without the need for the patient to travel anywhere. In their working paper, as part of the Southern African labour and development unit’s publication that looked at distance as a barrier to health care access, McLaren et al. defined the last kilometre in South Africa as less than 2 km from the nearest public clinic for about two-thirds of the population and less than 7 km for 90% of South Africans [20]. Their sub analysis for the Black African population group was motivated by the fact that they comprised 80% of the total population, 95% of the rural population and 95% of the poor; as such the rural- urban divide were calculated to be an average of 2 km versus 4 km, respectively, and the ratio of distance to the nearest public clinic to income was inversely proportional i.e. the poorest proportion of the population is the furthest away from the nearest health care facility as compared to more affluent people [20]. Yet in South Africa, the last kilometre delivery of medicines is not formally part of the health system (at the time of the study before 2020). Indeed, home delivery of medicines was restricted to isolated case; where community health workers have delivered medicines to immobile patients [21], and patient driven interventions consisted of local social entrepreneurs who charged a fee for collecting medicines on behalf of patients [18, 22].

The evidence for last kilometre delivery of medicines is primarily derived from postal services models, which might utilise a courier service either directly to the patient [23] or to their post box [24], and, in most instances, is applicable to patients with health insurance [25]. Abdulaziz et al. defined mail order pharmacy services as a “[health] system level intervention for improving access to chronic illness medications by making medications available to patients without the need to travel” [23] (Pg 1069). In the United States of America (USA) mail order pharmacies have been correlated with better adherence in patients with chronic diseases, but it is unclear if selection bias (patients who choose this option have better adherence than those who do not) could be a contributory factor [26]. Barriers to the use of mail order services include difficulties with using the ordering system, medicine order accuracy; concerns about mailbox security, longer waiting time (late delivery and running out of medicines), and restrictions on cold chain items (especially for post box delivery) [23, 24]. Facilitators for such services include mitigating travel to, and waiting time, at the pharmacy. Medical insurance schemes might also offer kickbacks to patients as it is cost-saving; a free month’s supply and longer (90 to 100 days) supply of medicines [24]. To our knowledge, there was no evidence of a formal last kilometre medicine delivery service in the South African public sector at the time of our data collection.

This study investigated patients’ preferences for a last kilometre medicine delivery service model. The preferences of patients who access their chronic medicines from public sector primary health care facilities in a poor, high-density housing area of the Cape Town Metropole, South Africa were investigated.

## Methods

### Study design and setting

This exploratory non-randomised quantitative study was conducted over 4 weeks between February 2018 and March 2018 at four Cape Town public primary health care facilities situated in low income and high-density housing (including formal and informal) underserved urban communities. These communities are socio-economically disadvantaged and are exposed to social determinants of ill-health namely, poor access to basic services, high crime rate, rising unemployment, food insecurity, poverty, along with a high prevalence of non-communicable diseases [27]. The facilities provide free health care services including medicines to the communities.

In terms of chronic care (for NCDs), at the time of data collection, these facilities employed chronic clubs and multi-month prescribing as DSD options for chronic patients. The chronic club is a systematic and comprehensive chronic disease management system implemented at primary care facilities for ambulatory chronic patients. The process entails an appointment booking system to guarantee a consultation either with a nurse (for minor observations and acute care) or doctor (to manage potential risks and review prescribed therapy), a register to monitor and evaluate patient health outcomes, and group health education sessions offered either via a nurse or health promoter [28].

### Target population

The target population were patients located in the pharmacy waiting area of the facility who were collecting any type of chronic medication for treatment or prevention of any condition (including HIV, non-communicable diseases and family planning). Patients were included in the study if they collected their chronic medication on a continuous basis (either monthly, 2 monthly or 3 monthly), and were over 18 years of age.

Patients were excluded from the study if they were less than 18 years of age or if they were collecting medication for an acute condition which did not require them to collect medicines from the pharmacy on a regular basis.

### Data collection

An independent, trained data collector administered a structured questionnaire to patients waiting in the queue at the pharmacy waiting area at each public sector health facility. The data collector who was fluent in English and other local languages including isiXhosa handled language queries, spent 1 week at a facility, then proceeded to the next one. The sample size was limited by the time available for data collection, willingness of the participants to participate in the study and the study exclusion criteria. On average, it took patients 15 min to complete the questionnaire.

The questionnaire was designed to capture socio-demographic information and preferred characteristics of a last kilometre medicine delivery service. The socio-demographic information such as age, gender, employment status, frequency of medicine collection, distance from home to clinic and pharmacy waiting time was collected. The characteristics of a medicine delivery service included general interest in a delivery service, willingness to use a mobile application to order medicines, preference for delivery location, and, willingness to pay for the delivery service including specifying the fee.

### Statistical analysis

Data was captured on an Excel sheet. Descriptive statistics was employed to present socio-demographic information of the study participants. The factors that influenced patients’ preferences for certain characteristics of a medicine delivery service were also investigated. The Chi-square test was implored to determine the association or relationship between socio-demographic variables and variables describing preferences associated with characteristics of a last kilometre medicine delivery service. Where the assumptions of Chi-square test were violated, the Fishers exact test was used. The Bonferroni post-hoc test was used to adjust for multiple associations and determine where the significance existed. A *p*-value of 0.05 or less was considered statistically significant. Statistical analyses were performed using IBM SPSS version 23. The statistical post hoc power of association (Chi-square test) was determined retrospectively based on a medium effect size (0.3) and 2 degrees of freedom using G*Power version 3.1.9.2. A power of ≥80% was considered desirable [29].

### Ethical approval

The study was approved by Pharma-Ethics (Pty) Ltd. (reference: 171018671). Permission to access the health facilities was obtained from the sub-district deputy director, Pharmacy services, Metropole District Health services. Participants were approached in the pharmacy waiting areas of facilities, they were informed about the study and if they agreed to participate, were asked to sign a written informed consent form. Participation was voluntary and participants had the right to withdraw their participation from the study at any time and without giving any reason. Neither identifiable data nor confidential health information was collected. No questions were asked about specific health issues or any other sensitive information. The data collected was treated with utmost confidentiality.

## Results

Of the 116 patients who participated in this study, two-thirds were female (69.8%) and 46 years or older (68.1%) (Table 1). Three-quarters (75%) of participants were unemployed. The majority of participants (79.3%) collected their medication on a monthly basis. More than half of participants lived within less than 1 km radius to the clinic (56%) and reported a waiting time of more than 3 h (55.2%).

**Table 1:** Socio-demographic information of participants (*n* = 116)

Variable	Sample size (percentage)
**Study site**	
Site A	24 (20.7)
Site B	36 (31.0)
Site C	31 (26.7)
Site D	25 (21.6)
**Age group**	
18 to 45 years	37 (31.9)
46 to 60 years	45 (38.8)
Over 60 years	34 (29.3)
**Gender**	
Male	35 (30.2)
Female	81 (69.8)
**Employment status**	
Employed	29 (25)
Unemployed	87 (75)
**Frequency of medication collection**	
Monthly	92 (79.3)
Every 2 months	16 (13.8)
Every 3 months	8 (6.9)
**Distance from home to clinic**	
Less than 1 km	65 (56)
1 to 5 km	33 (28.4)
More than 5 km	18 (15.5)
**Pharmacy waiting time**	
0 to 3 h	52 (44.8)
More than 3 to 6 h	40 (34.5)
More than 6 h	24 (20.7)

### Participant preferences associated with a last kilometre medicine delivery service

**Fig. 1:**
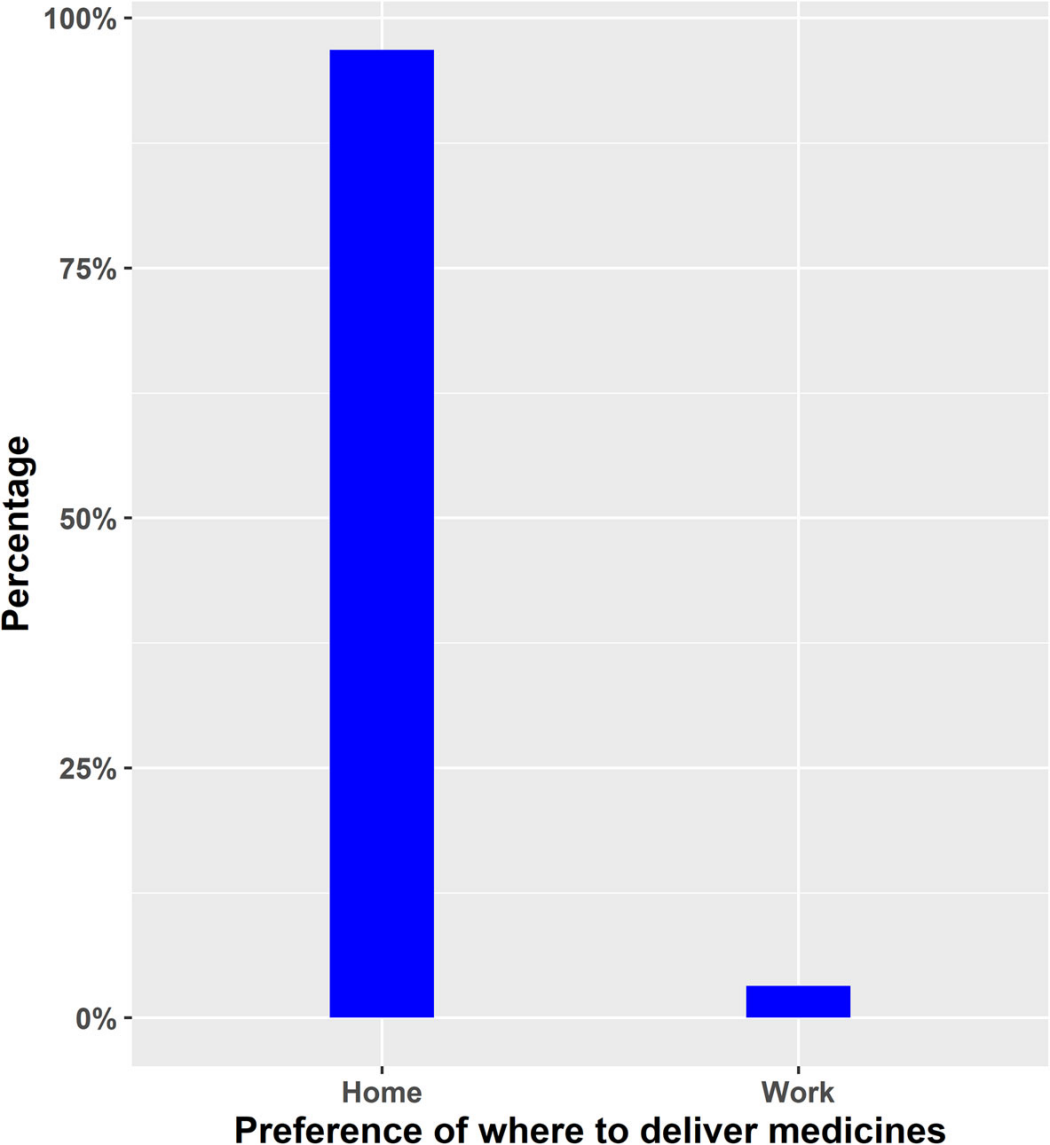
Participants’ preference for location of medicine delivery (*n* = 116)

**Fig. 2:**
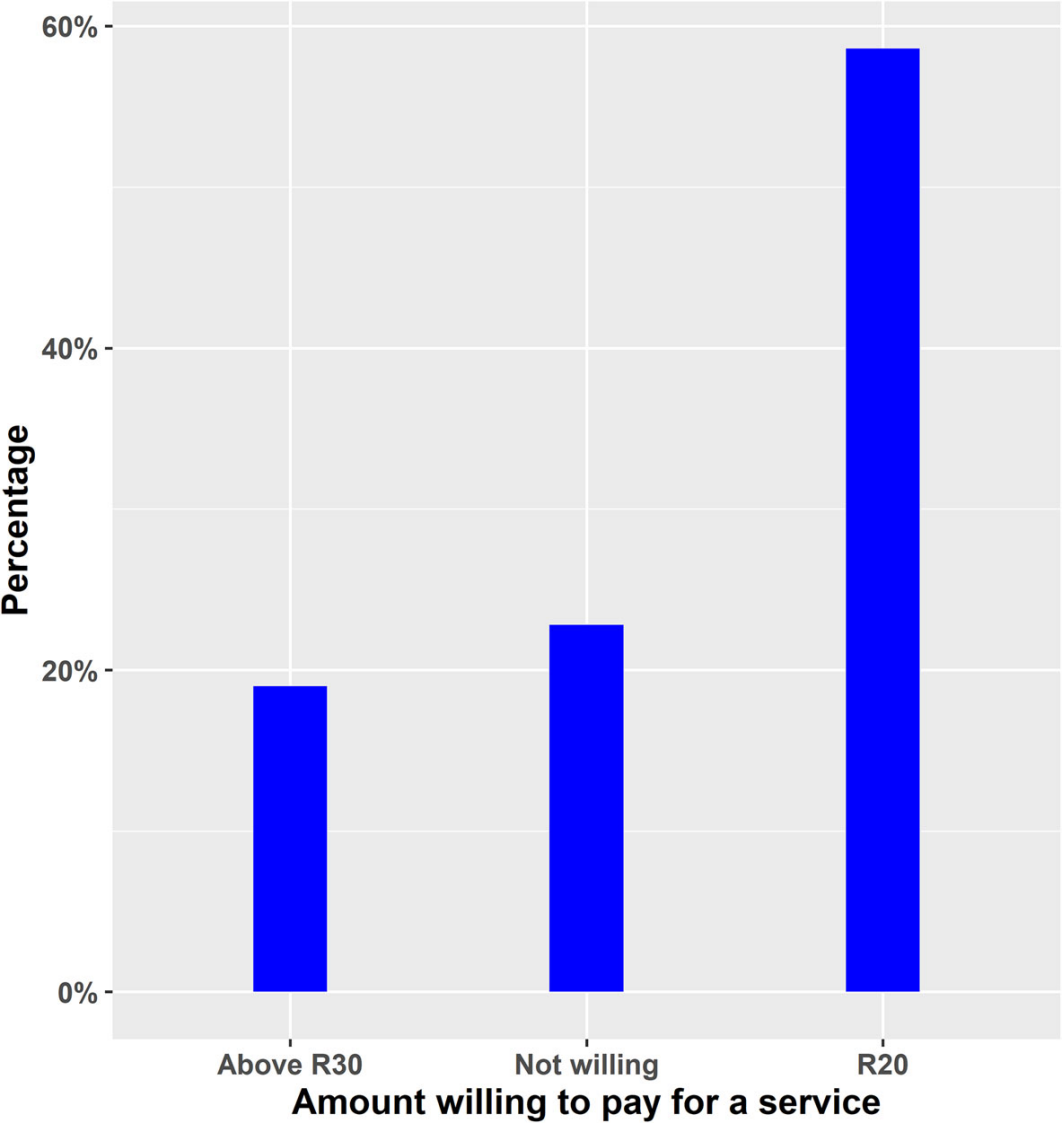
Participants (*n* = 116) willingness to pay for the mobile delivery service

Participants’ preferences relating to the characteristics of a medicine delivery service are presented in Table 2. Most participants were interested in the medicine delivery service (80.2%), and were willing to use a mobile application for ordering medicines (84.5%). Almost all (96.8%) chose to have medicines delivered to their homes (Fig. 1). In addition, more than three quarters (77.6%) of the participants indicated a willingness to make a payment, with the majority (58.6%) opting to pay 20 ZAR (USD 1.4) for the service (Fig. 2).

**Table 2:** Participant responses for preference towards a medicine delivery service (*n* = 116)

Delivery service preference variables	Response	
	Yes N (%)	No N (%)
Interested in medicine delivery service	93 (80.2)	23 (19.8)
Willing to use mobile application for ordering medicines	98 (84.5)	18 (15.5)
Willing to pay for the service	90 (77.6)	26 (22.4)

*N* = sample size

### Influence of participant’s socio-demographic situation on preference for medicine delivery service

The calculated statistical power using medium effect size of 0.3 and 2 degrees of freedom was 83.3%.

The interest in the medicine delivery service was significantly influenced by age (x^2^ = 40.6, *p* < 0.001), employment status (x^2^ = 58.7, *p* < 0.001), distance from home to clinic (x^2^ = 29.9, p < 0.001) the frequency of medicine collection (x^2^ = 7.5, *p* = 0.024) and pharmacy waiting time (x^2^ = 10, *p* = 0.007). Being 18–45 years was associated with an increased number of patients who were not interested in the medicine delivery service. Staying within 1 km from the clinic and waiting for more than 6 h at the pharmacy were associated with an increased number of patients who indicated an interest in the medicine delivery service. Having employment was associated with a low number of patients who showed an interest in the medicine delivery service. Collection of medicines once a month was significantly associated with a low number of patients who were interested in the medicine delivery service while collection of medicines every 2 months was associated with high number of patients.

Willingness to use a mobile application to order medicines was significantly influenced by age (x^2^ = 21.3, *p* < 0.001), employment status (x^2^ = 25.3, *p* < 0.001) and distance from home to clinic (x^2^ = 10.5, *p* = 0.005). Being 46 to 60 years and living within less than a 1 km radius to the clinic were associated with an increased number of patients willing to use a mobile application to order medicines. Additionally, age between 18 to 45 years and being employed were associated with a low number of patients willing to use a mobile application to order medicines.

Participant preference relating to location of medicine delivery was significantly influenced by employment status (x^2^ = 29.3, *p* < 0.001, power = 89.8%) and distance from home to clinic (x^2^ = 14.4, *p* = 0.001, power = 83.3%). Being employed was associated with a low number of patients who preferred their medicines to be delivered at home. Nevertheless, staying within less than 1 km radius from home to clinic was associated with a high number of patients who preferred that their medicines to be delivered at home.

The willingness to pay for the delivery service was significantly influenced by gender (x^2^ = 58.4, *p* < 0.001) age (x^2^ = 40.6, *p* < 0.001) and distance from home to clinic (x^2^ = 6.4, *p* = 0.042). Being female was associated with an increased number of patients willing to pay for the delivery service. The age group 18–45 years was associated with a low number of patients who were willing to pay for the delivery service. However, being 60 years and above was associated with increased number of patients willing to pay for the delivery service. Staying within a 1 to 5 km radius from home to clinic was associated with a high number of patients willing to pay for the delivery service.

The amount of money that patients were willing to pay for the delivery service was significantly influenced by gender (x^2^ = 41, *p* < 0.001) and employment status (x^2^ = 31.8, *p* < 0.001). Willingness to pay ZAR 20 (USD 1.4) was significantly associated with a high number of female patients while willingness to pay ZAR 30 (USD 2.1) and above was significantly associated with a high number of male patients. Willingness to pay ZAR 20 (USD 1.4) was significantly associated with a high number of participants who were not employed. However, being employed was associated with an increased number of patients willing to pay ZAR 30 (USD 2.1) and above.

## Discussion

This study determined the preferences associated with a last kilometre medicine delivery service from patients queuing for their chronic medicines in a pharmacy waiting area in public sector health facilities. Generally, interest in a last kilometre medicine delivery service was high (80.2% of participants were interested). This finding concurs with other South African literature which attested that although the formal health system does not offer the last kilometre / home delivery service, isolated cases in which community health workers delivered medicines to immobile patients were identified [21] and those patients who resorted to paying social entrepreneurs a service fee to collect medicines for them [18]. Further endorsement was reported in an ART study which showed the highest preference among patients to reduce the number of clinic visits [17].

Most participants in this study were older than 45 years (68.2%), indicating that our sample of patients would lean towards those being treated primarily for NCDs, as disease prevalence generally increases with an increase in age [3]. Similarly, Bobrow et al., [30] reported an average age of 54.7 years in their adherence study among hypertensive patients in Cape Town. The group that may be underrepresented in this sample might include patients with HIV which tend to be more prevalent in younger (aged 15–49 years) individuals in South Africa [31]. Indeed, a systematic review into interventions to improve treatment adherence and access to HIV care in sub-Saharan Africa found that less than 10% of participants were older than 50 years [32].

In general participants from the younger age group (18–45 years) were not interested in a medicines delivery service, When this is further correlated with preferences associated with characteristics of a medicine delivery service in terms of age, the younger age group (18–45 years) was not interested in using a mobile device for the ordering of their medicines and not willing to pay for this service. Conversely the older age groups showed significant associations with regards to willingness to use a mobile device to order medicines (46–60 years) and willingness to pay for a delivery service (older than 60 years). Similarly, literature agrees that postal delivery of medicines is associated with older individuals [23, 25].

The gender distribution among participants showed a female majority (69.8%), which seems to be a common finding in South Africa when recruiting participants from health facilities. In a pre-ART study, a similar Fig. (69%) of female participants was noted [12], while in an adherence trial among hypertensive patients, 28% male participants [30] enrolled. Literature agrees that women, traditionally, have better health seeking behavior than men and would more frequently seek medical help [33]. In particular, objectively measured health status for Black African women was reported to be worse than men, whereby women have been found to be significantly more obese and hypertensive than men [20], which may also explain the gender differences that exist in their health seeking behaviour. On the surface, there was no association between wanting a mobile delivery service in terms of gender. However, preference for certain characteristics of a medicine delivery service in terms of the willingness to pay for the service and the amount participants were willing to pay, start to reveal a more nuanced narrative that speaks to gender dynamics in South Africa, which is discussed further later in this section.

One quarter (25%) of participants in this study was employed, which is similar to a formal employment rate (24%) among participants from a South African study conducted in an urban high-density housing area, including informal and low cost housing with high rates of poverty [12]. Notably, South Africa’s general unemployment rate was reported at 27.1% (first quarter of 2018) [34]. Surprisingly being employed was associated with a low number of participants interested in a medicine delivery service. This finding tends to contradict other studies that found that time away from work was a major consideration related to medication adherence and that implied that employed participants would want services that minimises their time away from work [35]. It is also problematic in trying to compare these finding to research into postal delivery services because these research participants were more likely to have medical insurance [25], whereas our participants accessed free public healthcare services.

Most participants in this study collected their medicines on a monthly basis from the pharmacy (79.3%). Interest in last kilometre medicine delivery was negatively associated with receiving medicines on a monthly basis and positively associated with collecting medicines on a 2 monthly basis. Similarly, evidence from postal delivery studies agreed that patients who opted for medicine delivery services usually received multi-monthly prescriptions [23–25]. In addition, patients allocated to the chronic clubs at facilities are generally classified as 'stable', which means that their chronic condition is well controlled and they need less frequent care than patients who are not controlled and need more frequent follow up.

More than half of participants lived within a 1 km radius of the health facility (56%) which confirmed that most patients who made use of the clinic also lived in the clinic’s catchments area. Interest in last kilometre medicine delivery was positively associated with living within 1 km radius of the clinic. Additionally, in the South African context, the probability that people who live within 1 km of the clinic will have transport options from their home is unlikely, because South African public transport accessibility is generally between 10 to 30 min walking time from home [36], the significance of which might be augmented if the participant has difficulty to walk (immobility), a consideration among the elderly [21]. However, 15.5% of participants lived more than 5 km away from the clinic, which might indicate that they fell outside the clinic catchment area. Interestingly, these participants did not have any significant preference for or against any aspect of a medicines delivery service. This might indicate that such individuals have specific reasons for accessing care further away from home. Overall, these findings are similar to postal delivery literature from developed countries, which also did not find any associations between distance from the pharmacy and the likelihood to prefer mail delivery of medicines [25].

Participants reported a large variation in waiting time at the clinic, which ranged from less than 1 h to more than 6 h. In this paper the concept of waiting time refers to overall waiting time at the health facility (including waiting time at reception to get the patient files, waiting time for consultation, and waiting time at the dispensary). This is in accordance with South African policy documents such as the Ideal Clinic initiative, where waiting time is referred to as an overall figure which covers the total amount of time spent at the health facility [37]. The waiting times in this study is similar to waiting times recorded in another African study by Hardon et al. [11] which reported averages of 4 h or more (Botswana), 6 h (Tanzania), and 5 h (Uganda) waiting time in ART clinics [11]. A more recent study that conducted a process evaluation of the fidelity of implementation of the integrated chronic disease management model [38], found that the overall waiting time for patients receiving multi-month prescriptions varied between 2 and 5 h in four South African clinics [39]. According to the South African Ideal Clinic initiative, the target waiting time at any facility should ideally not exceed 3 h [37].

Not surprisingly, interest in making use of a medicine delivery service were positively associated with participants who reported a waiting time of more than 6 h at the clinic. Similarly, literature agrees that time saving benefits were one of the considerations for preference for postal medicine delivery [40]. Indeed, prolonged waiting time is not only an issue in South African facilities, but have been the reason for many health service innovations noted globally [41, 42].

### Participants’ preferred characteristics associated with a last kilometre medicine delivery service

The characteristics of a medicines delivery service that was explored included ordering of medicines on a mobile application, delivery location, willingness to pay for the service and amount participants were willing to pay for the service.

Most participants (84.5%) indicated their willingness to use a mobile device to order their medicines. An adherence study amongst adult hypertension patients in a public health facility in Cape Town found that nearly all participants owned their own cell phone, which supports the viability of using a mobile application for ordering medicines [30]. The Bobrow et al. [30] study also showed that support delivered via text messages could improve medicine collection and may have a small reduction in systolic blood pressure control as compared to routine care over 12 months [30]. However, solid evidence for using mobile technology in health care interventions is scarce [43].

In this study, willingness to use a mobile device to order medicines was positively associated with an age of 46 to 60 years, staying within 1 km radius of the clinic, and, negatively associated with ages of 18 to 45 years and being employed. The finding that participants between 46 and 60 years were interested to use a mobile device for ordering of medicines, partly contradicts literature that anticipated that elderly groups might be excluded from services that employ mobile ordering of medicines, because of the assumption that the elderly might not know how to use mobile phones [41]. Yet evidence from postal delivery services, mainly used by older populations, showed that mobile notifications and reminders were facilitating factors for uptake of this service [24]. Indeed, evidence seems to agree that adherence interventions using mobile technology that was effective for older individuals (average age 54.3 (11.5) years) with hypertension in the Bobrow et al. [30] study was different from interventions that was effective for younger individuals on ARVs [44]. These differences need to be taken into consideration when designing interventions across age groups.

Most participants (96.8%) preferred that their medicines be delivered at home. Delivery location preference to the home was positively associated with living within a 1 km radius from the facility, whereas employed participants preferred delivery to their workplace.

More than three quarters of participants (77.6%) were willing to pay for a medicine delivery service. This finding concurs with literature where chronic patients in Cape Town used pre-existing informal home delivery models since 2012 [18]. The willingness to pay for this service was positively associated with being female, being 60 years and above and living between 1 to 5 km from the facility, and negatively associated with the age group between 18 to 45 years. Literature investigating patient costs initially focussed on transport and loss of income due to time being away from work [10, 11]. Distance from the clinic would invariable be directly related to transport costs, it thus makes sense that people living further from the clinic would be more willing to pay for this service as they would be paying for transport anyway. Indeed, a study from Africa concluded that transport costs are an important reason why patients on ART failed to collect their medicines [11].

The finding that females where significantly more willing to pay for a medicine delivery service may allude to both direct and indirect costs involved for them to access a facility. As such, recent literature investigating patient costs included caring and capacity costs, in addition to transport costs and loss of income [12]. This might connect to the finding in this study where the female participants were more willing to pay for a delivery service, which could be related to their caring responsibility (children or other family members) and dependence on a carer before attending a clinic, thereby requiring more planning effort and incurring indirect expenses to access a clinic than men might have had.

The majority (58.6%) of participants showed a willingness to pay ZAR 20 (USD 1.4) for the service. Similarly, Magadzire et al. [18] reported that service fees for home delivery of medicine ranged from ZAR 10.00 to 20.00 (USD 1.00 to 2.00) between 2012 to 2014 and also speculated that these fees would offset normal transport costs [18]. Willingness to pay ZAR 20 (USD 1.4) was significantly associated with a high number of participants who were not employed. However, being employed was associated with an increased number of patients willing to pay ZAR 30 (USD 2.1) and above. Females were willing to pay ZAR 20 for delivery and males were willing to pay ZAR 30 or more.

The gender difference in the amount to be paid might be illustrative of the larger gender dynamics in South Africa. Despite, a strong legal framework in respect of gender equality and women’s rights, on the ground discriminatory practices, social norms and persistent stereotypes often shape inequitable access to opportunities, resources and power for women and girls. The most serious gender-related challenge includes unacceptable levels of gender-based violence [45]. In addition, women recorded a higher unemployment rate, lower absorption rates and lower labour force participation rates compared to their male counterparts [34]. Indeed, research on gender-based household compositional changes and implications for poverty in South Africa showed that female headed households were more vulnerable to lapse into poverty over time [46].

### Implications for a last kilometre medicine delivery service

In terms of adding the delivery of medicine as a component of pharmaceutical service delivery, the traditional face to face dispensing which include efficacy and safety monitoring and counselling associated with pharmaceutical care becomes a grey area. This has been noted as a professional concern in postal delivery research, yet evidence suggests that patients appear ready for this service [40]. Indeed, adherence research for mail order pharmacies suggests that patients opting for this service are more likely to be adherent and whether this is due to selection bias is unsure [26].

In terms of current medicine delivery initiatives in South Africa’s public health system the 2017 amendment of Good Pharmacy Practice (GPP) guidelines stipulated minimum standards for the collection and delivery of medicines to patients from a community or institutional pharmacy [47]. These rules stipulate that all efforts must be made to enable access to counselling by a pharmacist, and, when the patient is not available written instructions must be furnished by the pharmacist, which should include the patient’s details, information regarding the correct use of the medicine and a patient information leaflet (PIL) [27].

Although the role of the pharmacist is clear in the GPP; concerns about the proper implementation of these rules have been noted in literature that explored the perspectives of stakeholders’ based in the public health care system regarding novel community based medicine distribution models for improved access to chronic medicines in South Africa [18]. This study revealed tension between stakeholders (loosely divided between frontline health care workers and management level) based on expanding access and implementing policy. Although not to compromise service quality, some stakeholders called for policy that are context specific and pragmatic taking in account the limited resources and a balance between access to medicines versus strict adherence to the rigid standards set out by professional bodies [18]. Indeed, literature on the (sub-standard) implementation of policy in South Africa is prevalent and is in large part attributed to the high workload placed on frontline health care workers. In particular, audits based on the Ideal Clinic standards and GPP in public health care facilities in the Eastern Cape Province of South Africa have shown sub-par pharmaceutical care across the board [48].

More specifically, concerns regarding a lack of patient counselling inherent in community based distribution models have been noted [18]. One such model includes the previously mentioned delivery of medicines by community health workers [18, 21]. This model is an example of task shifting that has been used extensively in resource constrained environments to ease workload pressure from health care professionals to mid-level workers (such as pharmacist’s assistants or lay heath care workers primarily involved in adherence counselling) [18]. Although community health workers did emphasise the importance of taking medicines, this was not a common practice [21]. Additionally community health workers are linked to the facility through a nurse supervisor and not directly to the pharmacist, which limits their access to medicine-related information [21].

A possible counter measure to the concern of lack of counselling by a pharmacist or other individuals distributing medicines is addressed in the GPP which indicates the provision of written information including a PIL accompanying the medicines. However, package inserts and PILs are legally required to be written in English and one other official language (most the other language is Afrikaans), which imply that many patients would receive the written information in their second or third language [49].

In addition to medicines information, the maintenance of the integrity of medicine is also included in the GPP. Concerns that have been raised in a commentary written about the social entrepreneur delivery model included the security and integrity of the medicines during the transportation process [22]. As such, theft of the medicines that occurs during transportation has been reported [22]. This is especially common for anti-retroviral medicines such as efavirenz, which is used for recreational purposes. In addition the integrity of the medicines with respect to temperature control during transportation needs to be ensured [22].

In terms of sustainability of a home medicine delivery service, the two scenarios currently described in literature, which includes delivery of medicines via community health workers and social entrepreneurs has an element of complementary sustainability. Community health workers are employed via non-governmental organizations that are funded by government and /or external donor funding. Whereas, the social entrepreneurs initiative is funded by the patients themselves. An advantage of the social entrepreneur initiative is that it is a community-led pharmaceutical entrepreneurial initiative, which shows community empowerment and reduces reliance on government resources. However, from government’s perspective, literature indicates divergent views regarding these paid services. Provincial/senior managers had strong reservations about paying for services at primary health care level and feared patient exploitation, while some frontline health care practitioners claimed that this service was demand driven and should remain an option for patients as they felt the fees offset indirect costs to access a free service [18]. However, literature reported that some facilities where a fee for service was charged were mandated to seize this service, which illustrated the power of government to stop these services [18].

Most recently, as part of the response to the COVID-19 epidemic in South Africa, the Cape Town metropole health services decided to institute formal home delivery of medication to reduce the risk of COVID-19 in chronic patients [50]. This programme utilised and combined all of the current community distribution models, but relied heavily on home delivery of medicines by community health workers. A subsequent analysis of this initiative raised concerns related to a significant perceived change in the scope of practice of community health workers that might detract from their more important focus on broader primary health care, community diagnosis, routine household assessment, health promotion and disease prevention, which might become overshadowed by medicine delivery. Additionally, the sustainability of the initiative also relies on effective communication and coordination between role-players and managers for which a track record is yet to be established [50]. A permanent roll out of such a medicine delivery service as an additional task might walk the line of exploitation of community health workers and add to their already challenging working conditions [51].

Still, a last kilometre medicine delivery service as a DSD model in general provides an alternative for patients from current group-based community models, because it ensures privacy and seems to be stigma-neutral. Indeed, postal delivery services have shown to benefit patients in terms of its “assumed anonymity” in relation to sensitive health issues [25, 40]. In addition, a study that investigated a group-based community adherence club found that the assumed peer support aspect intended through the group seemed to be overestimated, especially in large urban areas, which tended to reduce the primary advantages of this model as essentially reduced waiting times and clinic visits [52].

Limitations of the study include the small sample size and inability to prospectively calculate the sample size and power due to funding restrictions. In addition, most studies used for comparison were studies investigating HIV and not NCDs and it is uncertain if these two will correlate due to the difference in stigma associated with conditions and the average age of the study populations. Another limitation was that neither the medical condition nor medications were recorded during data collection, also described elsewhere [25], thus our findings are less relatable to literature that specifically investigated specific conditions.

## Conclusion

This study found that chronic patients’ preferences for a last kilometre home delivery service should target patients older than 45 years and offer home delivery within a 5 km radius of a health facility. However, some of the socio-demographic characteristics that were less predictable or did not agree with literature were for those individuals that had employment and were living more than 5 km from the facility. This might indicate that these populations have other motivations that influence their adherence to chronic medicines and further studies focusing on these sub-populations are recommended. The significance of this study was exploring the patients’ preferences for a relatively novel last kilometre medicine delivery service in an uninsured community and developing country context. As such, these findings add to the evidence-base for delivery of medicine (often referred to as postal delivery services in developed contexts) and align with findings from South Africa of community-led interventions that provides economic development opportunities (social entrepreneurs) in underserved communities linked to the health system.

## Supplementary Information

**Additional file 1.**

**Additional file 2.**

## Data Availability

The datasets used and/or analysed during the current study are available upon reasonable request of the corresponding author.

## Abbreviations

ART: Antiretroviral therapy
DSD: Differentiated service delivery
GPP: Good pharmacy practice
HIV: Human immunodeficiency virus
NCD: Non-communicable disease
PIL: Patient information leaflet
USA: United States of America
USD: United States dollar
ZAR: South African rand
