# Supplementary material for: Access to chronic medicines: patients’ preferences for a last kilometre medicine delivery service in Cape Town, South Africa
Source: BMC Fam Pract. 2021 Feb 22;22:43. doi: 10.1186/s12875-021-01392-1 (PMC7899209; doi:10.1186/s12875-021-01392-1)
Supplement: Supplementary file 2 — Additional file 2. [file 12875_2021_1392_MOESM2_ESM.pdf]

Participant survey

Criteria to proceed with survey

Do you pick up medication on a monthly/regular basis (chronic care, family planning)?

- Yes
- No

What is your age?

- Less than 18 years
- 18 to 20 years
- 21 to 30 years
- 31 to 45 years
- 46 to 60 years
- Over 60 years

If respondent answers Yes to Question 1, and are over 18 yrs old the researcher continue the survey. If not, end survey.

Start of Survey

Name of the health care facility

Gender of Participant

- Male
- Female

Language of Participant
………………………

Are you employed?

- Yes
- No
- Other…………

How many kms do you live from the clinic?

- Less than 1km
- 1 to 5 km
- More than 5 km

How often do you pick up medication?

- Once a month
- Once every 2 months
- Once every 3 months
- Other (Please explain) ……..

What is the average amount of time you spend waiting to pick up medication?

- 0 to 1 hour
- 1 to 3 hours
- 4 to 6 hours
- More than 6 hours

Would you be interested in a service that picks up and delivers your medication to you?

- Yes
- No
- Other (please explain)………

Where would you like the delivery to be sent?

- Home
- Work
- Campus
- Other (please explain)……….

Would you use a mobile app to order medicines if it saved you having to pay for transport?

- Yes
- No

What would you be willing to pay for the pick up and home delivery service?
R………..
